# Advancing the Frontiers of Neuroelectrodes: A Paradigm Shift towards Enhanced Biocompatibility and Electrochemical Performance

**DOI:** 10.3390/polym16111457

**Published:** 2024-05-22

**Authors:** Qin Wang, Yiyang Liu, Baolin Zhang, Jianghui Dong, Liping Wang

**Affiliations:** 1 School of Intelligent Medicine and Biotechnology, Guangxi Engineering Research Center of Digital Medicine and Clinical Translation, Guilin Medical University, Guilin 541004, China; qinwang997@163.com; 2 Guangxi Key Laboratory of Optical and Electronic Materials and Devices, College of Materials Science and Engineering, Guilin University of Technology, Guilin 541004, China; 1020210185@glut.edu.cn (Y.L.); baolinzhang@ymail.com (B.Z.)

**Keywords:** superparamagnetic iron oxide nanoparticles, Dimyristoyl phosphatidylcholine, neural recordings

## Abstract

The aim of this study is the fabrication of unprecedented neuroelectrodes, replete with exceptional biological and electrical attributes. Commencing with the synthesis of polyethylene glycol and polyethyleneimine-modified iron oxide nanoparticles, the grafting of Dimyristoyl phosphatidylcholine was embarked upon to generate DMPC-SPION nanoparticles. Subsequently, the deposition of DMPC-SPIONs onto a nickel–chromium alloy electrode facilitated the inception of an innovative neuroelectrode–DMPC-SPION. A meticulous characterization of DMPC-SPIONs ensued, encompassing zeta potential, infrared spectroscopy, X-ray photoelectron spectroscopy, and X-ray diffraction analyses. Evaluations pertaining to hemolysis and cytotoxicity were conducted to ascertain the biocompatibility and biosafety of DMPC-SPIONs. Ultimately, a comprehensive assessment of the biocompatibility, electrochemical properties, and electrophysiological signal acquisition capabilities of DMPC-SPION neuroelectrodes was undertaken. These findings conclusively affirm the exemplary biocompatibility, electrochemical capabilities, and outstanding capability in recording electrical signals of DMPC-SPION neuroelectrodes, with an astounding 91.4% augmentation in electrode charge and a noteworthy 13% decline in impedance, with peak potentials reaching as high as 171 μV and an impressive signal-to-noise ratio of 15.92. Intriguingly, the novel DMPC-SPION neuroelectrodes herald an innovative pathway towards injury repair as well as the diagnosis and treatment of neurological disorders.

## 1. Introduction

Neuroelectrodes connect the human body to external devices, recording the electrical signals emitted by neurons and transmitting them to external devices to explore brain functioning, repair motor–sensory impairments, and diagnose and treat neurological diseases [1,2]. The transmission of electrical signals from neuroelectrodes is achieved through ion-electron conversion channels at the interface between neuroelectrodes and biological tissues. However, implanted electrodes inevitably cause inflammation, are predisposed to astrocyte formation, cause electrode failure, and can even disrupt the blood–brain barrier [3,4]. It is evident that neuroelectrodes need to have good mechanical, biocompatible, and electrochemical properties, and the continuous improvement of performance has become an important challenge in this field.

Currently reported to improve the performance of neuroelectrodes are two commonly used methods; one is to prepare a porous structure on the electrode sites to increase the effective surface area and improve the performance of the electrodes; the second is to modify the surface of the electrodes, modifying materials, drugs and biomolecules, etc., to change the electrode’s mechanical properties, electrical properties, cellular behavior, etc. [5]. Commonly used modified electrode site materials have superior mechanical and electrical properties, which facilitates rapid electron and ion migration and improves the electrochemical performance of the neuroelectrode. Modified materials are categorized into three types: carbon-based materials and metal oxides and conductive polymers. Carbon-based materials include glassy carbon, carbon fiber, carbon nanotubes, graphene, and others [6,7]. Metal oxides include iridium oxide; conductive polymers include polyaniline, polythiophene, and polypyrrole [8,9,10]. Cui et al. doped polystyrene sulfonate (PSS) into PEDOT to form PEDOT: PSS composites, which were coated onto the electrode surfaces, greatly increasing the effective area of the electrode–biological tissue interface and improving electrochemical performance [11]. Su et al. used excellent multiplicative performance with TiO_2_-coated self-contained flexible electrodes [12]. Heim et al. deposited carbon nanotubes (CNTs) and conductive polymers on electrodes to substantially improve sensitivity and electrical stimulation efficiency [13].

Superparamagnetic iron oxide nanoparticles (SPIONs) below 10 nm have good superparamagnetism, excellent magnetic field response, and have been widely used in biomedical applications such as magnetothermia, MRI, bioseparation, catalysis, etc. [14]. SPIONs have good dispersibility and biocompatibility after surface treatment. Dimyristoylphosphatidylcholine (DMPC) has similar components of cell membranes, consisting of the tails of two long double carbon chains, the middle part of the hydrophobic hydrocarbon chain, and the head consisting of amphipathic ions containing phosphate and choline. DMPC is extremely hydrophilic and biocompatible [15,16] and does not trigger immune responses [17]. SPIONs encapsulated with DMPC and PEG drastically reduced the immune response triggered by cellular phagocytosis [18,19,20]. Therefore, in this study, the hypothesis of modifying DMPC on SPIONs was proposed to improve the mechanical, biocompatible, and electrochemical properties and to construct novel neuroelectrodes with superior performance.

**Figure 1:**
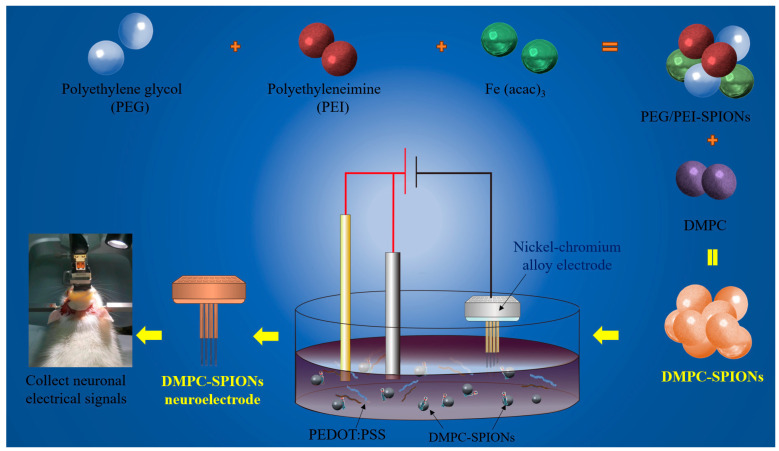
Preparation of DMPC-SPION neuroelectrode.

Therefore, PEG/PEI-SPIONs were synthesized using a modified polyol thermal decomposition method, and DMPC was grafted onto PEG/PEI-SPIONs to form nanoparticle DMPC-SPIONs. The DMPC-SPIONs were deposited onto a nickel–chromium alloy electrode to construct a novel DMPC-SPION neuroelectrode (Figure 1). The nanoparticles were characterized by determining the hydrated particle size, zeta potential, TEM, infrared spectroscopy, XRD, and XPS, the biological properties of the nanoparticles were tested by hemolysis assay, cytotoxicity assay, and cellular uptake assay, and finally the biocompatibility, electrochemical properties, and capability in recording electrical signals of the DMPC-SPION neuroelectrodes were evaluated.

## 2. Materials and Methods

### 2.1. Materials

The reagents used in the preparation of PEG/PEI-SPIONs were polyethylene glycol (PEG) sourced from Shanghai Aladdin Biochemical Technology Co., Ltd., Shanghai, China, PEI, iron (III) acetylacetonate (Fe(acac)_3_) procured from Shanghai Aladdin Biochemical Technology Co., Ltd., Shanghai, China, and acetone and toluene solution acquired from Shantou Xilong Chemical Co., Ltd., Shantou, China. DMPC from Shanghai Yuanye Biotechnology Co., Ltd., Shanghai, China, was added in the preparation of DMPC-SPIONs. During the modification of the nerve electrode, the deposition solution needs to be configured, and poly (styrene sulfonic acid) (PSS) acquired from Shanghai Aladdin Bio-Chem Technology Co., Ltd., Shanghai, China, and 3,4-ethylenedioxythiophene (EDOT) from Shanghai Aladdin Bio-Chem Technology Co., Ltd., Shanghai, China, were used here.

### 2.2. Preparation of DMPC-SPIONs for Neuroelectrodes

#### 2.2.1. The Preparation of PEG/PEI-SPIONs

In this study, PEG/PEI-SPIONs and DMPC-SPIONs were synthesized based on preliminary experiments using a modified polyol thermal decomposition method [21].

A quantity of 15 g of PEG harmoniously coalesced with 0.3 g of PEI from the aforementioned distributor, in a 50 mL three-neck flask. The amalgamation underwent thorough stirring using a stir bar within a well-controlled argon environment. Upon the temperature within the flask reaching a high 80 degrees Celsius, a further addition of 0.7 g of Fe(acac)_3_ was introduced. Subsequently, the temperature was escalated to an impressive 260 °C and allowed to reflux for a long duration of 60 min. The resultant mixture was subsequently subjected to a thorough triple rinsing procedure employing a toluene solution followed by an acetone rinse from the same supplier. The employment of ultrasonic cleaning further augmented the purification process.

After the culmination of the meticulous washing procedure, the acetone was meticulously eliminated, and deionized water was judiciously administered to disperse the sample, facilitating the fruition of PEG/PEI-functionalized superparamagnetic iron oxide nanoparticles (PEG/PEI-SPIONs). This assemblage of nanoparticles was conscientiously deposited in a refrigerated environment, precisely at a temperature of 4 °C, awaiting subsequent application.

#### 2.2.2. The Preparation of DMPC-SPIONs

A total of 10 mg of DMPC was mixed with a solution of 10 mL PEG/PEI-SPIONs at a concentration of 1 mg/mL. The mixture then reacted for 5 h at 4 °C in a temperature-controlled shaker (TS-200B, Shanghai Tiancheng Experimental Instrument Manufacturing Co., Ltd., Shanghai, China) with a rotational speed of 100 r/min. Subsequently, the sample was subjected to 2–3 rounds of size exclusion chromatography using LS magnetic bead columns (MT Technology GmbH, Ingolstadt, Germany) to remove free DMPC. Finally, the samples of DMPC-SPIONs were successfully fabricated.

#### 2.2.3. The Preparation of DMPC-SPION Neuroelectrodes

First, configure the deposition solution for electrodeposition: Combine PEG/PEI-SPIONs and DMPC-SPIONs in a collective volume constituting 1%, and dilute them with deionized water until reaching a final volume of 30 mL. Incorporate 0.18 g of PSS and disperse it within the solution utilizing ultrasound for a duration of 10 min. Additionally, infuse 0.03 g of EDOT into the solution, and agitate it utilizing ultrasound for a period of 30 min to procure the deposition solution.

Utilize an electrochemical workstation (CHI660A, Shanghai Lichangbangxi Instrument Technology Co., Ltd., Shanghai, China) for electrochemical deposition. PEDOT: PSS with good electrical conductivity and certain adhesion properties enables DMPC-SPIONs to attach more firmly to the electrodes [22]. Establish a three-electrode system featuring a calomel electrode as the reference electrode, a platinum wire electrode as the counter electrode, and a nickel–chromium alloy electrode as the working electrode. During the multi-potential step, designate one step with a potential of 1 V and a step time of 300 s. Following the deposition process, respectively acquire the PEG/PEI-SPION neuroelectrodes and the DMPC-SPION neuroelectrodes.

### 2.3. Performance Testing

#### 2.3.1. Material Performance Testing

In this investigation, the water hydrodynamic dimensions and zeta potential of the nanomaterials were assessed employing a state-of-the-art nanoparticle size analyzer and zeta potential analyzer (Nano ZS, Malvern Instruments Ltd., UK). The morphology of the nanoparticles was observed through the utilization of a transmission electron microscope (TEM) (TF20, FEI Company, USA). The chemical properties of the nanoparticle surface were examined using advanced Fourier-transform infrared spectroscopy (FTIR) (H-7650, Nicolet Instrument Corporation, USA) and X-ray photoelectron spectroscopy (XPS) (K-Alpha X, Thermo Fisher Scientific Inc., USA). The phase of the nanoparticles was elucidated utilizing X-ray diffraction (XRD) (Ultima VI, Rigaku Corporation, Japan). The concentration of the Fe element in the nanomaterials was quantified by means of inductively coupled plasma atomic emission spectroscopy (Optima 7300DV, PerkinElmer Inc., USA).

#### 2.3.2. Hemolysis Assay

A volume of 1 mL of freshly drawn murine blood was collected in an Eppendorf tube containing a minute quantity of anticoagulant solution (obtained from Shanghai Aladdin Biochemical Technology Co., Ltd., Shanghai, China). Subsequently, the tube was placed within a state-of-the-art, high-capacity tabletop centrifuge (Jidi-5d, Guangzhou Jidi Instrument Co., Ltd., Guangzhou, China) and subjected to centrifugation at 1000 rpm for a duration of 10 min to eliminate the supernatant. The resultant red blood cells were then meticulously washed with a PBS solution and subsequently re-suspended in an identical PBS solution, thus yielding a homogeneous red blood cell suspension. For the subsequent step, a total of 50 μL of the aforementioned red blood cell suspension was thoroughly mixed with equal volumes of solutions containing PEG/PEI-SPIONs and DMPC-SPIONs at varying concentrations of 5, 10, 25, 50, 75, and 100 μg/mL, respectively. In parallel, a control group consisting of a PBS solution was employed as the “negative control” (NC), whereas pristine water served as the “positive control” (PC). The resulting mixtures were incubated under controlled conditions at a temperature of 37 °C for a duration of 60 min and thereafter subjected to centrifugation at 1000 rpm for an additional 10 min. The supernatant was further extracted and transferred into a 96-well plate, followed by the quantification of its absorbance at a wavelength of 590 nm using a cutting-edge, multifunctional microplate reader (Spark, Decan Shanghai Laboratory Equipment Co., Ltd., Shanghai, China). The calculation of the hemolysis percentage entailed applying Formula (1) [23].

Hemolysis% = (X − Y)/(Z − Y) × 100%(1)

In the above equation, X is the absorption value of the experimental group, Y is the absorption value of the negative control group, and Z is the absorption value of the positive control.

#### 2.3.3. Cellular Cytotoxicity

Cellular cytotoxicity was assessed utilizing the MTT (3-(4,5-dimethylthiazol-2-yl)-2,5-diphenyltetrazolium bromide) assay [24]. Each well of a 96-well plate was inoculated with 1 × 10^4^ cells and incubated in 100 μL of RPMI 1640 complete medium (Thermo Fisher Scientific (China) Ltd., Shanghai, China) in a CO_2_ incubator (Thermo Fisher 370, Thermo Fisher Scientific (Suzhou) Instruments Co., Ltd., Suzhou, China) for a duration of 24 h. Subsequently, the RPMI 1640 medium was aspirated and substituted with PEG/PEI-SPIONs or DMPC-SPIONs at incremental concentrations spanning from 5 to 200 μg/mL. Following additional incubation periods of 24, 48, and 72 h, 10 μL of MTT solution (5 mg/mL) (Beijing Solarbio Science & Technology Co., Ltd., Beijing, China) was administered to each well and allowed to incubate for an additional 4 h. Afterward, 100 μL of a triplicate solution (comprising 10% sodium dodecyl sulfate, 5% isopropanol, and 0.01 M HCl) was added overnight to facilitate the dissolution of the resultant formazan crystals. The absorbance at 570 nm was subsequently quantified using a multifunctional microplate reader to ascertain cell viability.

#### 2.3.4. Experiment of Cellular Uptake

In every well of a 6-well plate, 5 × 10^5^ cells were seeded and cultured with approximately 1 mL of RPMI 1640 complete medium for 24 h. Following the incubation period, the RPMI 1640 complete medium was replaced with 200 μg/mL of PEG/PEI-SPIONs and DMPC-SPIONs for 12 h to promote uptake. Subsequently, the cells were centrifuged in centrifuge tubes, and the supernatant containing the nanoparticles was removed carefully. The cells were then rinsed 2–3 times with PBS solution until the supernatant appeared colorless. Finally, the cells were fixed with 2.5% glutaraldehyde (Beijing SinoLaneTech Co., Ltd., Shanghai, China).

#### 2.3.5. Cell TEM Preparation

The fixed cells were washed three times with 0.1 M PBS (pH = 7.0) for 15 min per wash. Afterwards, the cells were fixed with a 1% osmic acid solution (Shanghai Aladdin Bio-Chem Technology Co., Ltd., Shanghai, China) for 1–2 h. Following the removal of the osmic acid solution, the cells were rinsed three times with 0.1 M PBS (pH = 7.0) for 15 min per wash. Subsequently, the cells were dehydrated using a series of ethanol gradients (30%, 50%, 70%, 80%) for 15 min per gradient, followed by 90% and 95% acetone solutions for 15 min each. The cells were then dehydrated twice using a pure acetone solution for 20 min per dehydration. Ultimately, the samples were treated with a mixture of embedding agent and acetone (*V*/*V* = 1/1) at room temperature for 1 h. The resin blocks were sectioned into ultra-thin sections measuring 70–90 nm using an ultramicrotome (UC7, Leica Instruments GmbH, Wetzlar, Germany) and collected on copper grids. The sections were stained with uranyl acetate (Guangzhou Zhongjing KEJI Technology Co., Ltd., Guangzhou, China) for 8–15 min, followed by staining with lead citrate (Guangzhou Zhongjing KEJI Technology Co., Ltd., Guangzhou, China) for 8–10 min. Finally, the samples were observed using a transmission electron microscope (H-7650, Hitachi High-Tech Corporation, Japan).

#### 2.3.6. Intracellular Fe Content Test

The fixed cells were centrifuged and rinsed with 0.1 M PBS (pH = 7.0). To lyse the cells, they were treated with a cell crusher and then heated in a water bath containing 0.3 mL concentrated hydrochloric acid and 1 mL nitric acid for 2 h. Following this, 0.1 mL of Triton X-100 was added, and the mixture was further heated for 1 h. Once complete cell lysis was achieved, the samples were diluted to a specific volume, and the cellular iron content was determined using ICP-OES (5110, Agilent Technologies, USA).

#### 2.3.7. Interfacial Biocompatibility of DMPC-SPION Neuroelectrodes

Platinum wire replacement electrodes depositing PEG/PEI-SPIONs and DMPC-SPIONs were bilaterally implanted into the hippocampal region of the mouse brain of AP: −2.06 mm, ML: 1.35 mm. Experiments were categorized into three distinct experimental groups: the blank control group, the PEG/PEI-SPIONs group, and the DMPC-SPIONs group. Each platinum implant possessed a specialized coating, one adorned with PEG/PEI-SPIONs and the other with DMPC-SPIONs. Upon the nineteenth day post-implantation, the hearts, livers, spleens, lungs, and kidneys of these rodents were systematically extracted and then meticulously prepared as refined pathological sections. These sections were imbued with the combination of Hematoxylin and Eosin (H&E) (esteemed Beijing Zhongshan Golden Bridge Biotechnology Co., Ltd. of Beijing, China) resulting in the crafted H&E staining pathology slides.

#### 2.3.8. CV Testing and Electrode EIS Testing of DMPC-SPION Neuroelectrodes

CV testing and electrode EIS testing were performed using an electrochemical workstation (CHI660A, Shanghai Li-Chen Bang Xi Instrument Technology Co., Ltd., Shanghai, China). As a result of the limited effective surface area of the Ni-Cr alloy electrode, substituting platinum wires or other metal wires can substantially broaden the deposition surface of the modifying substance. This enhances the ability to showcase its impact on the animal in vivo.

First, cyclic voltammetry testing was performed with a calomel electrode as the reference electrode, a platinum wire electrode as the counter electrode, and a DMPC-SPION neuroelectrode as the working electrode. The voltage–current curves were obtained by controlling the electrode potential to cycle in a triangular waveform over time at a certain scanning rate and measuring the current through the electrode surface. According to the area enclosed by the cyclic voltametric curve, the charge storage capacity of the electrode can be known, and an excellent charge storage capacity can ensure that the electrode can carry out safe and effective stimulation in vivo. In the test, the initial potential was set to be 0 V, the high potential to be 1 V, and the low potential to be 0 V. The starting scanning polarity was positive, and the scanning rate was 0.05 V/s, and the number of scanning segments was 3 segments.

Then, an electrochemical impedance (EIS) test was used to evaluate the neuroelectrode performance. EIS responds to the ability of the electrode to record signals, and the higher the electrode impedance, the lower the signal-to-noise ratio of the electrode-recorded signals. Measuring the AC impedance of the electrode can make an initial judgement on the conductivity of the electrode and can also guide the electrode preparation process so that the electrode can be further prepared minutely to reduce tissue damage during implantation. Generally, referring to the impedance value at 1 KHz to evaluate the electrode, the test can be started by setting the initial potential to the same as the open circuit potential after selecting the AC impedance in the experimental technique.

#### 2.3.9. Acquisition of Electrophysiological Signals from DMPC-SPION Neuroelectrodes

This study has obtained approval from the Animal Care and Experimentation Committee at the Shanghai Experimental Animal Center, with the distinguished serial number 20211201039.

**Figure 2:**
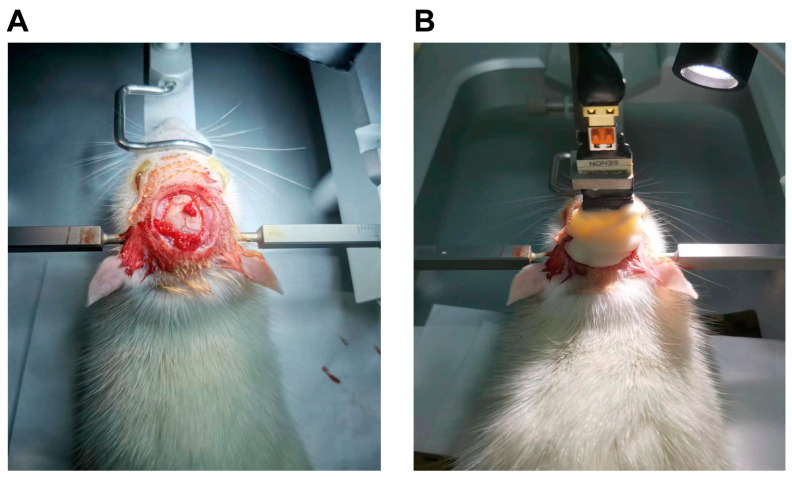
(**A**) Exposing the skull. (**B**) Neuroelectrode implantation.

Fifteen SD rats were randomly divided into three groups, a control group with pristine neuroelectrodes, a group with PEG/PEI-SPION neuroelectrodes, and a group with DMPC-SPION neuroelectrodes. First, general anesthesia was administered by an intraperitoneal injection of 1% sodium pentobarbital. Then, they were then meticulously secured onto a brain stereotaxic apparatus (69100, Shenzhen RWD Life Science Co., Ltd., Shenzhen, China), the length of the ear bars on both sides was adjusted to make them symmetrical, the tight ear bars were fixed, and then the upper incisors of the rats were fixed using a brain stereotaxic apparatus. Then, the skin above the skull of the rat was cut along the sagittal suture, posteriorly from the hindbrain and anteriorly to the forebrain, and the skin on both sides was propped up with hemostatic forceps. According to the intricate mouse stereotactic brain atlas, the precise target sites within the medial prefrontal cortex brain region were identified (AP −2.06 mm, ML 1.35 mm). The electrode base was carefully fixed on the gripper on the positioner, the angle was adjusted so that the direction of the electrode wire was perpendicular to the plane of the skull (Figure 2A), it was connected to the input of the signal acquisition system, and the electrode was slowly brought close to the center of the cranial window, and the earth wire on the electrode was tightly wrapped around the nearby screws. This was followed by a meticulous and rapid penetration through the soft meninges into the brain tissue and was finally fixed on the skull (Figure 2B).

Post-implantation, the electrodes were firmly secured in place within the brain using dental bone cement, ensuring complete coverage and stability. Upon awakening from anesthesia, the mice were housed individually in pristine cages, allowing for solitary recuperation. Neuronal signals were meticulously collected one week after the mice had fully regained their strength post-operation. These signals were derived from the intricate hippocampus of the mouse brain, utilizing a state-of-the-art multichannel neural acquisition processor operating at a frequency of 40 kHz and band-pass filtering in the range of 300–5000 Hz.

The acquired neuronal signals were then subjected to a rigorous analysis using an advanced in vivo multichannel electrophysiological acquisition system (KD-RHD1, Kedou (Suzhou)Brain-Computer Technology Co., Ltd., Suzhou, China).

#### 2.3.10. Statistical Analysis

All experiments were replicated at least three times, and the data are presented as the mean ± standard deviation. Statistical comparisons were conducted using a *t*-test. Statistical analysis was performed using Origin software 2018 (Origin Lab Corporation, Northampton, MA, USA). Significance levels were considered as * *p* < 0.05 and ** *p* < 0.01.

## 3. Results

### 3.1. Subsection

#### 3.1.1. Hydrated Particle Size and Zeta Potential

**Figure 3:**
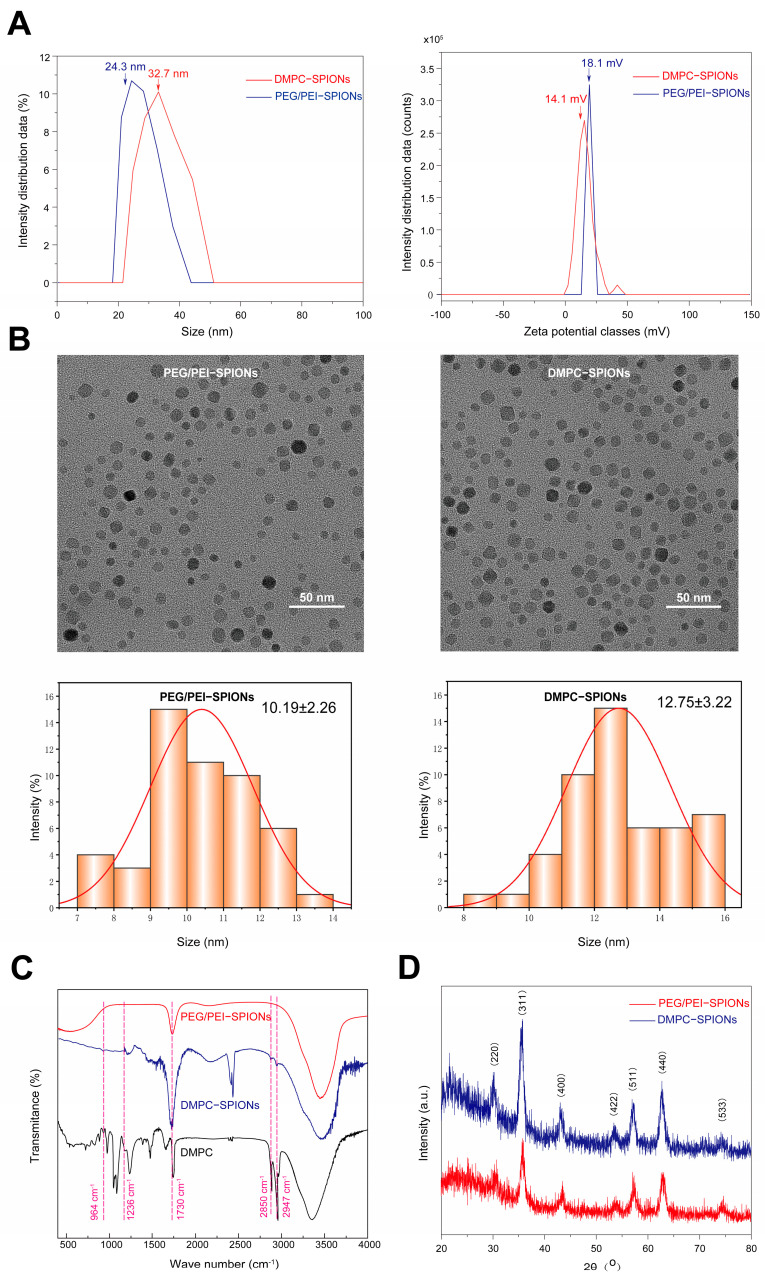
PEG/PEI–SPIONs and DMPC-SPIONs of (**A**) hydrated particle size and potential. (**B**) TEM image with particle size distribution. (**C**) FTIR image. (**D**) XRD image.

Figure 3A illustrates the hydrated particle dimensions of PEG/PEI-SPIONs and DMPC-SPIONs, measuring at 24.3 and 32.7 nanometers, respectively. The zeta potentials of PEG/PEI-SPIONs and DMPC-SPIONs are recorded at 14.1 and 18.1 millivolts, correspondingly. Owing to the influence of DMPC, both the hydrated particle size and zeta potential of PEG/PEI-SPIONs have undergone alterations, indicating the successful modification of DMPC on PEG/PEI-SPIONs.

#### 3.1.2. TEM Test

Figure 3B showcases the transmission electron microscopy images and particle size distributions of PEG/PEI-SPIONs and DMPC-SPIONs. Employing Image J 1.54i software, a sample of 50 nanoparticles was randomly chosen for analysis, revealing the average particle sizes of PEG/PEI-SPIONs and DMPC-SPIONs to be 10.19 ± 2.6 nm and 12.75 ± 3.22 nm, respectively. It is evident that these nanoparticles exhibit uniform dispersion and consistent sizes. The hydrated particle sizes were obtained through the dynamic light scattering (DLS) technique, which measures the hydrodynamic diameter of the nanoparticles, encompassing the particle core and the surrounding solvation shell or other molecules moving along with the particles. This measurement typically includes the nanoparticle core and its encompassing hydrated layer. Conversely, TEM analysis requires the drying of nanoparticles and examination under vacuum conditions. As it omits the hydrated layer, TEM results provide a more precise estimation of nanoparticles’ actual size, and TEM images offer visual information regarding particle distribution, size, and shape.

#### 3.1.3. FTIR Test

Figure 3C illustrates the FTIR spectra of PEG/PEI-SPIONs and DMPC-SPIONs. It is evident from the spectra that the absorption peak at approximately 964 cm^−1^ displays an asymmetric vibration, which can be attributed to the presence of CN^+^–(CH_3_)_3_, thus indicating the existence of DMPC and DMPC-SPIONs [25]. In contrast, no such absorption peak is observed for PEG/PEI-SPIONs. Notably, the absorption peaks of DMPC and DMPC-SPIONs are observed at approximately 1236 cm^−1^ and 1730 cm^−1^, respectively. These peaks correspond to the vibrations of the polar head group (PO^2−^) and C=O stretching. Conversely, these peaks are absent in the spectra of PEG/PEI-SPIONs. Consequently, it can be concretely confirmed that the successful modification of DMPC onto PEG/PEI-SPIONs has occurred based on these distinctive peaks [26]. And telescopic absorption by –C-H also occurs near 2930 cm^−1^ and 2850 cm^−1^. Similarly, the FTIR spectra of lipid-modified gold nanorods, as reported by Orendorff et al., also displayed the generation of an asymmetric vibration caused by CN^+^–(CH_3_)_3_ around 970 cm^−1^ [25]. Additionally, Giri documented the discovery of absorption peaks generated by –PO_4_^3−^ group vibrations at 1236 cm^−1^ during the process of phospholipid phosphatidylcholine modification on magnetite superparamagnetic nanoparticles [27].

#### 3.1.4. XRD Test

Figure 3D showcases the X-ray diffraction (XRD) patterns of PEG/PEI-SPIONs and DMPC-SPIONs. Distinct diffraction peaks emerge at 30.1°, 35.4°, 44.3°, 53.3°, 56.6°, 62.5°, and 74.0°, corresponding to well-defined crystal planes (220), (311), (400), (422), (511), (440), and (533), respectively. Remarkably, these findings align harmoniously with the PDF standard card for Fe_3_O_4_ (JCPDS 01-085-1436) [28]. Thus, it indicates that Fe_3_O_4_ serves as the principal crystalline phase in both PEG/PEI-SPIONs and DMPC-SPIONs. Extensive investigations have consistently demonstrated that the dominant crystalline phase in nanoscale SPIONs is Fe_3_O_4_.

#### 3.1.5. XPS Test

XPS analysis serves as a valuable technique for discerning the oxidation state information and elemental composition on the surface of nanoparticles. Consequently, it finds utility in investigating whether DMPC undergoes any modifications on the nanoparticles.

**Figure 4:**
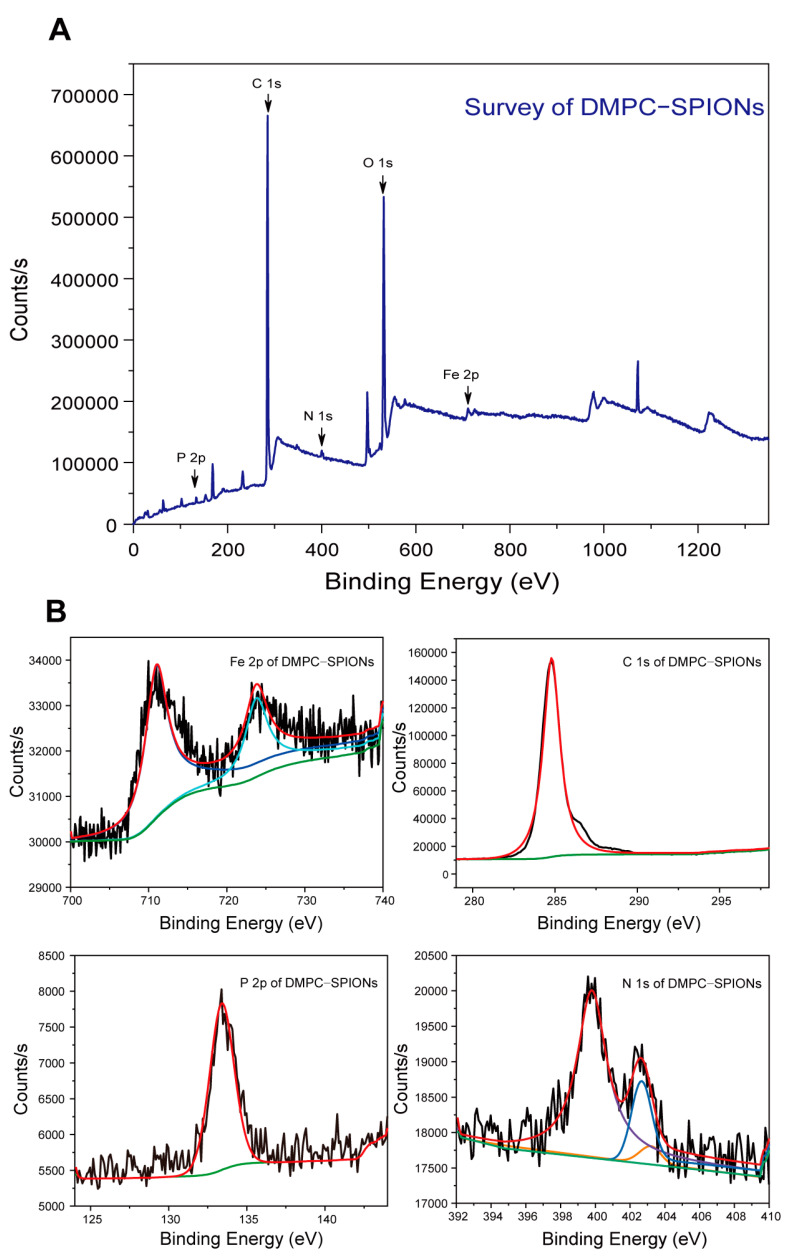
(**A**) XPS full spectrum. (**B**) XPS fine spectra of DMPC-SPIONs.

As depicted in Figure 4A, the XPS full spectrum of DMPC-SPIONs reveals prominent peaks at energy levels of 134.10 eV, 286.00 eV, 400.00 eV, 530.00 eV, and 711.00 eV, which correspond to P 2p, C 1s, N 1s, O 1s, and Fe 2p, respectively. Figure 4B offers a more detailed examination of the spectra for DMPC-SPIONs. Within the finely resolved spectrum of Fe 2p, distinct fitting peaks emerge, assigned with precision to Fe 2p_3/2_ (711.7 eV) and Fe 2p_1/2_ (725.6 eV) states. Positioned at an energy level of 286.00 eV, the binding peak accentuates the presence of carbon (C 1s) within DMPC-SPIONs. Furthermore, the detection of a peak at 134.1 eV clearly signifies the involvement of phosphorus (P 2p) in the modified composition. Regarding the N 1s spectrum of DMPC-SPIONs, since three distinct functional groups exist, namely N-H, C-N-C, and N^+^-(CH_3_)_3_, it is evident that three clearly defined peaks emerge near energy levels of approximately 399.70 eV, 402.45 eV, and 403.00 eV. These collective findings unambiguously confirm the successful modification of DMPC onto PEG/PEI-SPIONs.

### 3.2. Hemolysis Assessment

In accordance with the esteemed criteria established by the Material Experiment Society, when the hemolysis rate falls below 2%, the material can be deemed non-hemolytic. A hemolysis rate ranging from 2% to 5% illustrates a state of mild hemolysis, whereas a hemolysis rate surpassing 5% signifies outright hemolysis. Ergo, when the hemolysis rate of a biomaterial remains under 5%, one can confidently assert that the said biomaterial boasts a high level of biocompatibility.

**Figure 5:**
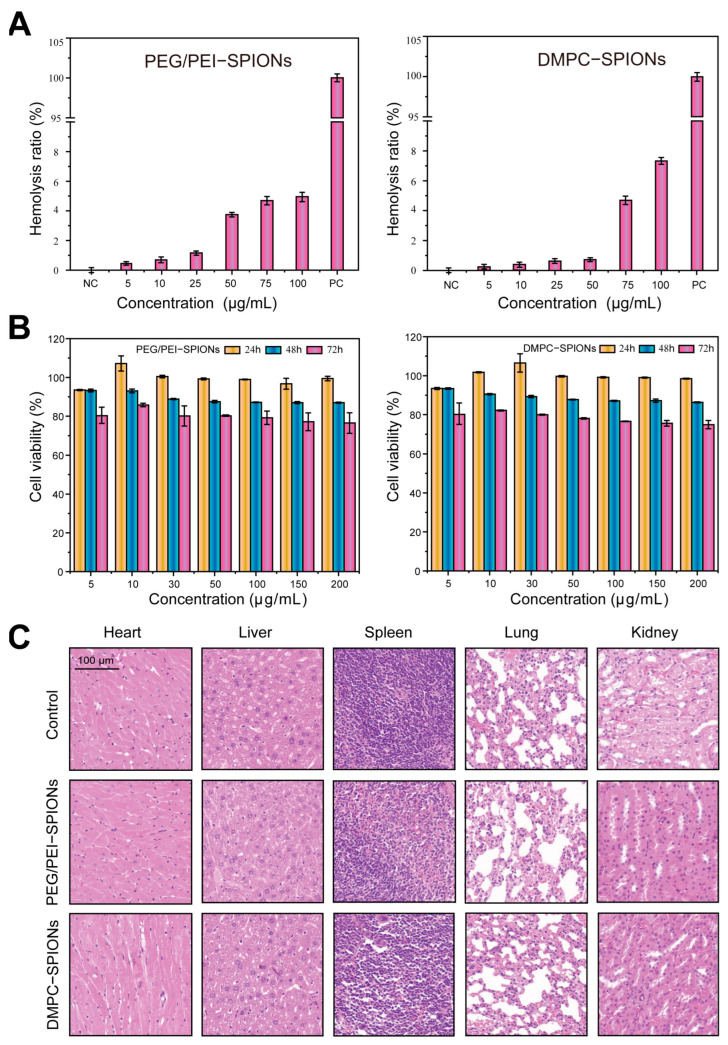
(**A**) Hemolysis rate. (**B**) Cytotoxicity. (**C**) Biocompatibility of major organs in mice.

The surface structure and properties of nanoparticles may have been altered after surface modification; in particular, the similar chemical structure of DMPCs to cell membranes makes them more likely to bind to receptors or molecules on the erythrocyte membranes, which may lead to the disruption of erythrocyte structure or function. As delineated in Figure 5A, as the concentration of the PEG/PEI-SPION solution ascends to 100 μg/mL, the hemolysis rate registers at 5%. Conversely, upon surpassing a concentration of 75 μg/mL in the DMPC-SPION solution, the hemolysis rate exceeds 5%. By consequence, it becomes clear that the PEG/PEI-SPION solution, when maintained at a concentration not exceeding 100 μg/mL, as well as the DMPC-SPION solution, when its concentration remains below 75 μg/mL, both exhibit only negligible hemolysis, thus signifying commendable biological safety.

### 3.3. Cell Toxicity Experiment

In order to further ascertain the biocompatibility of DMPC-SPIONs, PC-12 cells were selected as the cellular model, and the MTT assay was employed to evaluate the cytotoxicity of these two modified materials on PC-12 cells. The experimental findings are depicted in Figure 5B. Following a 24 h co-culturing period with these two materials, the cell viability surpassed 95%; after 48 h, it remained above 90% (cell viability exceeding 80% is deemed non-deleterious to cells). Henceforth, it can be deduced that both PEG/PEI-SPIONs and DMPC-SPIONs exhibit exceptionally low cellular toxicity and showcase remarkable biocompatibility.

### 3.4. Evaluation of Biocompatibility

In order to test the safety and effectiveness of the modified electrode interface on the organism, 14 days after the implantation of Pt replacement electrodes into mice, three groups of mice were dissected (control group, PEG/PEI-SPION electrode group, and DMPC-SPION electrode group), and the major organs, including the heart, liver, spleen, lungs, and kidneys, were collected to make pathologic sections.

The effects of the electrodes on the health of mice were investigated, and the results are shown in Figure 5C. The pathological sections of the major organs heart, liver, spleen, lung, and kidney in the PEG/PEI-SPION electrode group and DMPC-SPION electrode group were maintained in the normal histomorphology, and there was no obvious inflammation or organ damage, which proved that the PEG/PEI-SPION modified electrodes had good biocompatibility.

### 3.5. Cellular Uptake

Normally, cell membranes that are electronegative and positively charged nanomatrices will agglomerate with each other, and positively charged nanoparticles will be more susceptible to oxidative stress than negatively charged nanoparticles [29,30], and at the same time, positively charged DMPCs have a similar phospholipid structure to cell membranes, which makes it easier for SPIONs to enter the cells. Modifier flaking in modified electrodes is a common problem in implantation experiments. The confirmation of modifier shedding in experimental animals due to biological factors is necessary in short-term implantation experiments. Therefore, by co-culturing with PC-12 cells and conducting an ICP-OES test to determine the Fe content of the cultured cells, the enhancement effect of DMPC on the entry of SPIONs into the cells can be determined, and it can also provide a reference for the subsequent determination of the detachment of modifications due to cellular adsorption after the nanoparticle modification of the electrode.

**Figure 6:**
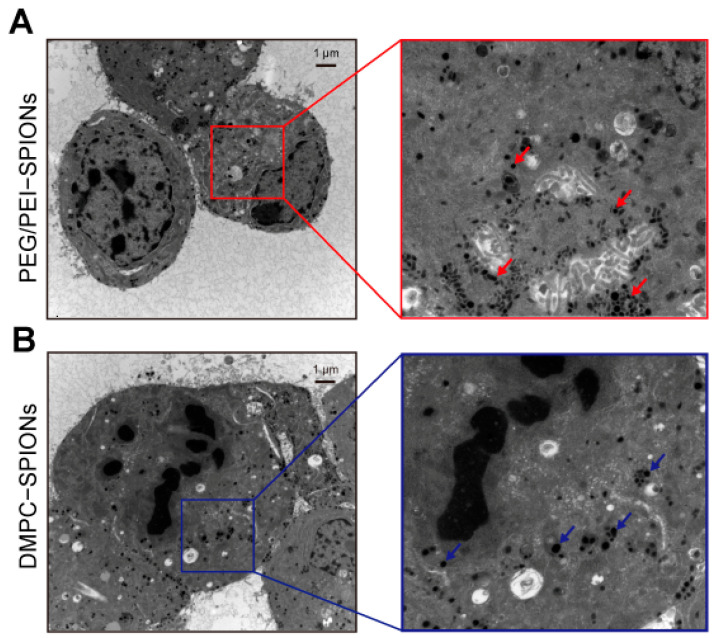
Distribution of nanoparticles within PC-12 cells. (**A**) PEG/PEI-SPIONs. (**B**) DMPC-SPIONs.

The PEG/PEI-SPIONs and DMPC-SPIONs were co-cultured with PC-12 cells for a duration of 12 h prior to undergoing TEM examination. The TEM image in Figure 6A illustrates the internalization of PEG/PEI-SPIONs by PC-12 cells over the course of 12 h, while Figure 6B depicts the uptake of DMPC-SPIONs by the same cells during the same timeframe. Upon conducting an analysis of the intracellular iron content subsequent to co-cultivation with PC-12 cells, a significant discrepancy emerged: the uptake of DMPC-SPIONs surpassed that of PEG/PEI-SPIONs by a factor of 1.8. This heightened affinity can be ascribed to the structural resemblance between DMPC and the phospholipid bilayer of the cellular membrane, thereby facilitating the entry of DMPC-SPIONs into the cellular milieu. Notably, it was observed that DMPC-SPIONs exhibited a heightened attraction towards negatively charged cellular membranes, consequently enhancing their cellular uptake. The introduction of DMPC onto nanoparticles to yield DMPC-SPIONs significantly bolstered endocytosis, thereby elevating their cellular uptake capabilities. Evidently, the data suggest that DMPC-SPIONs exhibit a greater propensity for cellular internalization compared to PEG/PEI-SPIONs.

**Figure 7:**
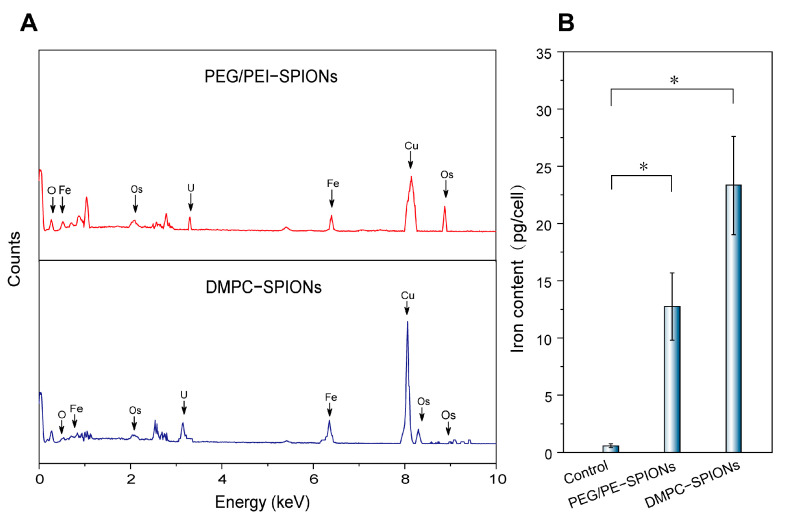
(**A**) EDS profiles of nanoparticles. (**B**) Intracellular content (pg/cell) (* *p* < 0.05).

In order to establish the nature of the dark clusters depicted in the TEM image as iron oxide nanoparticles, a validation process was undertaken employing the energy-dispersive X-ray spectrometer (EDS) which was integrated within the TEM instrument. The EDS spectrum, displayed in Figure 7A, revealed distinct X-ray spectra at 6.40 keV (Fe Kα) and 7.06 keV (Fe Kβ), indicative of the presence of iron. Moreover, it is worth noting that the peaks corresponding to Cu Kα, Os Kα, and U Kα emissions can be attributed to the employment of a copper grid and specific chemical agents utilized for tissue fixation and staining. These results provide unequivocal confirmation that the dense, black dots are composed predominantly of iron.

The ICP-OES test showed that the intracellular iron content was 12.74 pg/cell after 12 h of the co-culture of PEG/PEI-SPIONs with PC-12 cells, and the intracellular iron content was 23.35 pg/cell after 12 h of the co-culture of DMPC-SPIONs with PC-12 cells, as shown in Figure 7B and Table 1; the DMPC- SPIONs had approximately 1.8 times the intracellular iron content of PEG/PEI-SPIONs in the cells.

### 3.6. CV Testing and Electrode EIS Testing of DMPC-SPION Neuroelectrodes

**Figure 8:**
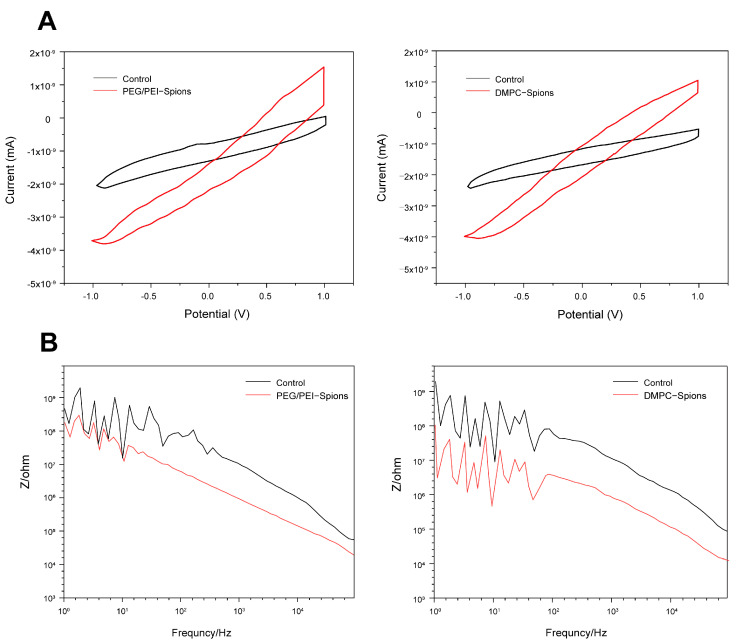
(**A**) CV curves and (**B**) EIS curves of neuroelectrodes.

Information such as the charge storage capacity of the electrodes can be obtained from the CV curves, as given in Figure 8A, where the modified Ni-Cr alloy electrodes were placed in phosphate buffer under the three-electrode system, the control is electrodes without any modifications, and the CV curves were measured at a sweep speed of 0.05 V/s. The charge storage of the electrodes after PEG/PEI-SPIONs and DMPC-SPIONs was significantly increased, which was calculated to be about 88.6% for the PEG/PEI-SPION modified electrodes and 91.4% for the DMPC-SPION modified electrodes. These results indicate that PEG/PEI-SPIONs and DMPC-SPIONs modified on electrodes can improve the capacitive performance of electrodes.

When the scanning rate is the same, the change in electrode charge can be judged by the area of the closed graph enclosed by the CV curve. As PEG/PEI-SPIONs and DMPC-SPIONs are continuously deposited onto the Ni-Cr alloy electrodes, the area constituted by the current–potential curves on the CV curves is constantly increasing, so that the electrode charge is improved, and also the impedance of the Ni-Cr alloy electrodes is also greatly reduced, which plays a positive role in the electrical conductivity of the electrode and facilitates the electrode for electrical stimulation and recording (Figure 8A).

This experiment not only demonstrated the successful deposition of PEG/PEI-SPIONs and DMPC-SPIONs onto Ni-Cr alloy electrodes by measuring the CV curves but also judged the charge storage capacity of the modified electrodes and explored the feasibility of the Ni-Cr alloy electrodes to collect neuronal electrical signals. The AC impedance of the modified PEG/PEI-SPIONs, DMPC-SPIONs, and unmodified Ni-Cr alloy electrodes was tested in phosphate buffer, respectively, as shown in Figure 8B. At 1 kHz, the electrode impedances of the modified PEG/PEI-SPIONs and DMPC-SPIONs were reduced to about 1 MΩ and 90 KΩ, respectively, which corresponded to an 11% and 13% reduction, respectively. Since this Ni-Cr alloy is homemade in the laboratory, there can be uneven electrode wire surfaces, sizes, etc., resulting in unstable impedance values in the low-frequency region.

### 3.7. Acquisition of Electrophysiological Signals from DMPC-SPION Neuroelectrodes

**Figure 9:**
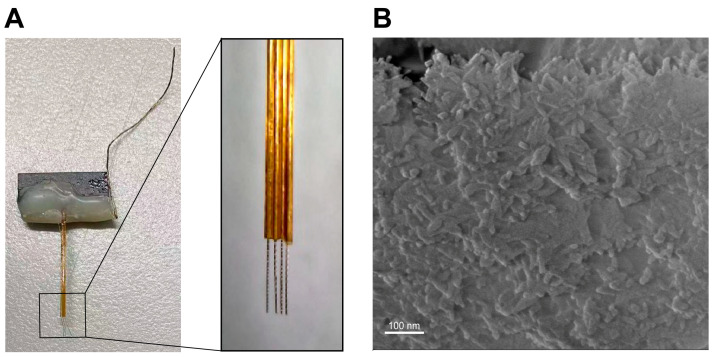
(**A**) Neuroelectrodes coated with DMPC-SPIONs. (**B**) Scanning electron microscope images of electrode surface modified with DMPC-SPIONs.

The depiction of the DMPC-SPION neuroelectrode utilized in this study is illustrated in Figure 9A, showcasing two electrode wires enveloped by a shared silicon core tube, collectively forming an eight-channel electrode that is affixed to the neural interface with epoxy resin. Following the deposition of DMPC-SPIONs onto the surface of the electrode wires, as exhibited in Figure 9B, the surface displayed an intricate, dense three-dimensional structure, enhancing the effective surface area of the electrodes. The presence of PEDOT: PSS further bolstered the attachment of the DMPC-SPIONs.

**Figure 10:**
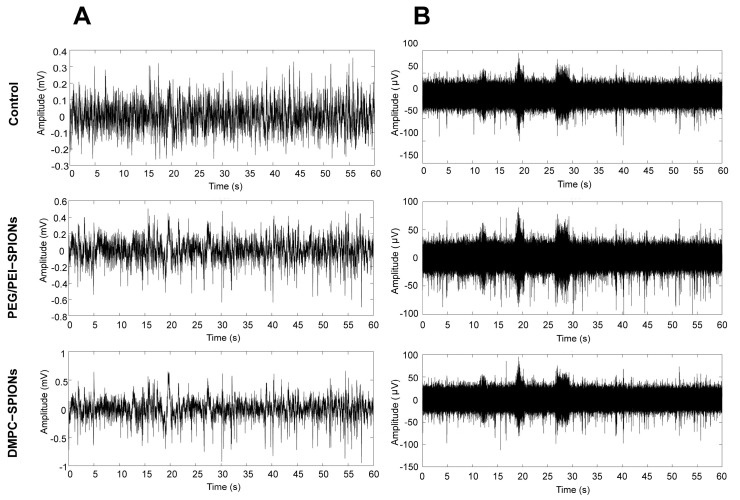
(**A**) Neuroelectrode acquisition of local field potential maps. (**B**) Neural signals after high-pass filtering.

The original signals of neurons recorded by the multichannel electrophysiology acquisition system are local field potentials (LFPs) and spikes, with neural signals sampled at a frequency of 40 kHz in rats and band-pass-filtered in the range of 300–5000 Hz to record LFPs and spikes, as shown in Figure 10.

The LFP represents the sum of postsynaptic potentials recorded by electrodes, reflecting the electrical activity of the target brain area and revealing the coordinated action of neurons in neural networks. The LFPs collected by neural electrodes are shown in Figure 10A. The potential fluctuations are smaller, lacking distinct troughs and peaks. Compared to the signals collected in the control group, the signals from DMPC-SPION neural electrodes exhibit significant fluctuations with larger peaks, improved signal quality, and better anti-interference capabilities.

Spikes represent the sum of action potentials discharged by multiple neurons recorded at the tip of the electrode. After high-pass filtering the signals, low-frequency noise is reduced. The signal intensity at the recording site of DMPC-SPION neural electrodes exhibits the highest vibration intensity and larger peaks, with signal fluctuations amplitudes greater than PEG/PEI-SPION neural electrodes and blank electrodes, and the peaks and valleys are more pronounced (Figure 10B). Additionally, there is no apparent regularity in the discharge time and periodicity of the signals.

**Figure 11:**
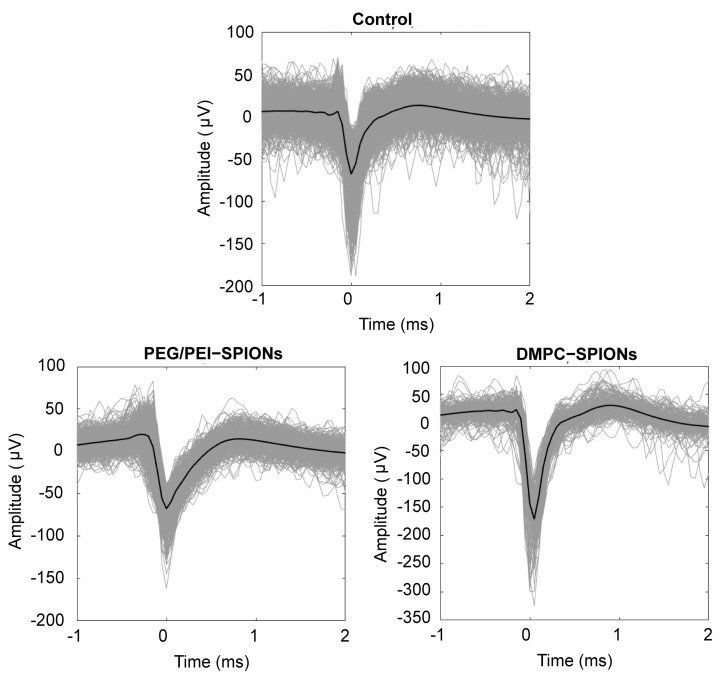
Neuroelectrode acquisition for 60 s to obtain waveforms of front potentials.

Subsequently, the acquired signals are subjected to threshold detection. When a signal exceeds the set threshold, it is marked as a spike, and the spike potentials further form spikes. The waveform of hippocampal neuron spike potentials collected for 60 s by neural electrodes is shown in Figure 11. The maximum amplitudes of the discharge signals of hippocampal neurons collected by the three groups of neural electrodes are approximately 55, 60, and 171 μV, respectively, with signal-to-noise ratios (SNRs) of 6.38, 7.21, and 15.92. It is evident that the DMPC-SPION neuroelectrode significantly enhances the efficiency of signal acquisition and exhibits a remarkable prowess in capturing neural signals.

## 4. Discussion

With the objective of addressing the scientific conundrum surrounding the biocompatible and electrochemical attributes of neuroelectrodes, a progressive investigation unfolded. Within the confines of this research, PEG/PEI-SPIONs materialized, appositely adorned with DMPC, thereby adorning the very foundation of Ni-Cr alloy electrodes. Ergo, an innovative specimen, the DMPC-SPION neuroelectrode, emerged from the experiment. Notably, this groundbreaking development demonstrates commendable biocompatibility. Moreover, it beholds exceptional electrochemical prowess, characterized by heightened charge storage capabilities coupled with reduced impedance levels and electrophysiological signal acquisition capabilities.

In this investigation, the utilization of PEG/PEI provided the essential functional groups for the modification of DMPC, thus resulting in the formation of DMPC-SPIONs. These DMPC-SPION particles exhibited uniform size distribution and displayed exceptional stability and dispersion properties. The surface charge of these nanoparticles was positively charged, which facilitated their interaction with biological tissues. It is worth mentioning that both the size and geometric characteristics of nanoparticles play a crucial role in their interaction with living organisms. For instance, gold nanoparticles exhibit varying interactions with endothelial cells depending on their morphology, with hollow nanoparticles showing a significantly lower uptake rate compared to rods and spheres [31]. Furthermore, the toxicity of gold nanoparticles is found to be size-dependent, with smaller particles around 1 nm being less toxic than those measuring around 15 nm [32].

Secondly, these fabricated DMPC-SPIONs exhibit minimal hemolytic activity and low cytotoxicity, possessing enhanced biocompatibility and safety. The surface of iron oxide nanoparticles, modified with PEG and PEI, abounds in amino and carboxyl groups, thereby improving biocompatibility. DMPC serves as a phospholipid that is modified on the surface of nanoparticles, facilitating their facile crossing of cellular membranes [33,34,35]. The surface structure and properties of nanoparticles may be altered after surface modification. In particular, the similarity of the chemical structure of DMPC to the cell membrane makes it more likely to bind to receptors or molecules on the erythrocyte membrane, which can lead to the disruption of erythrocyte structure or function.

However, with the incorporation of DMPC-SPIONs onto the electrodes, they exhibit a tendency to undergo cellular adsorption and subsequent detachment, leading to the deterioration of electrode performance. In our investigation, neuroelectrodes were fabricated by anchoring DMPC-SPIONs onto electrodes coated with PEDOT: PSS. The conductive polymer PEDOT: PSS was utilized to ensure a more durable adhesion of the modifications during the electrodeposition process. PEDOT: PSS consists of two constituents, specifically the hydrophilic PSS and the hydrophobic PEDOT, forming a core–shell-like interface that significantly enhances the stability of functional molecules on the electrode surface and fosters strong interfacial connections between electrode sites and neurons. Not only does it possess excellent electrical conductivity, but it also exhibits a certain viscosity that mitigates the detachment of the DMPC-SPIONs [22]. Gupta et al. augmented the electrode with gold nanoparticles in conjunction with PEDOT: PSS, resulting in a sixfold increase in the sensitivity of the electrode [36]. Hao et al. fabricated GH-PP-Pt/GCE electrodes by composing graphene hydrogel with PEDOT: PSS, followed by the in situ electrodeposition of Pt nanoparticles, thereby attaining excellent stability and immunity to interference [37]. Badi utilized tungsten oxide, gold nanoparticles, and PEDOT: PSS to compose a ternary composite electrode (PEDOT:PSS-WO_2_-Au), which exhibited exceptional electrochemical performance, still maintaining approximately 92% capacitance retention after 5000 cycles of operation [38].

**Figure 12:**
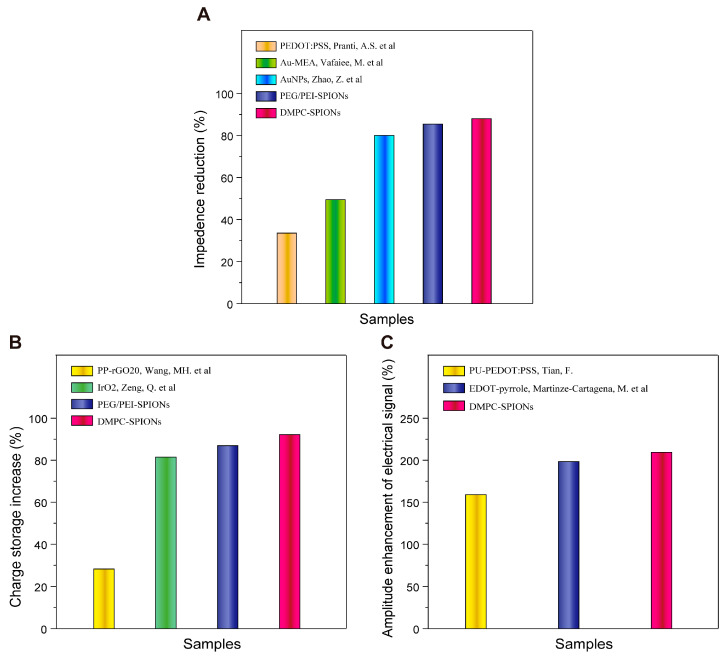
(**A**) Comparison of EIS curves for different modifiers. (**B**) Comparison of CV curves for different modifiers. (**C**) Comparison of capacity to capture physiological electrical signals [6,39,40,41,42,43,44].

Subsequently, the neuroelectrode composed of DMPC-SPIONs crafted in this study demonstrates outstanding electrochemical efficiency. The charge of the DMPC-SPION neuroelectrodes was heightened by approximately 91.4%, while the impedance was significantly reduced by an impressive 89%. These findings are consistent with those documented in the scientific literature, as depicted in Figure 12. Pranti ingeniously improved the performance of the gold electrode through the application of a PEDOT: PSS coating, resulting in a substantial 32.8% decrease in impedance [39]. Vafaiee adeptly engineered a gold multi-electrode array (Au-MEA), further enhanced by the integration of carbon nanotubes into the array, leading to a notable 50% reduction in impedance [40]. Conversely, Zhao skillfully deposited AuNPs on the gold electrode, resulting in a remarkable 81% decrease in impedance [41]. On the other hand, the electrode modified with PP-rGO20 by Wang et al. exhibited a modest 29.8% increase in charge [6], whereas Zeng et al.’s use of an iridium oxide-modified electrode recorded a substantial 83% amplification in charge [42].

Therefore, it is apparent that by modifying the electrode sites with PEDOT: PSS and functional molecules, the electrochemical effectiveness of the electrode can be significantly enhanced. The inclusion of DMPC-SPIONs effectively enhances not only the electrical conductivity but also elevates the electrical charge of the electrode. This unique feature opens up possibilities for stimulating neural activity under the influence of a magnetic field, making it a highly promising approach for developmental research.

Furthermore, this study revealed that the magnitude of the signals obtained from the DMPC-SPION neuroelectrodes surpassed that of the control group and the PEG/PEI-SPION group. The spike electrical signals exhibited peak amplitudes of approximately 55 μV, 60 μV, and 171 μV, respectively, with SNRs of 6.38, 7.21, and 15.92, respectively. The electrodes exhibited heightened sensitivity to the bioelectrical signals, boasting superior signal-to-noise ratios, nearly a twofold augmentation in signal amplitude, and an approximate doubling in the SNR as compared to the control group. This indicates a potential enhancement in the capacitance between the electrode and biological tissues due to the modification of the electrodes by the DMPC-SPIONs, thereby enhancing the quality of electrophysiological signals. For example, Tian enhanced the electrode with PU-PEDOT: PSS hydrogel, and a subsequent analysis of spike electrical signals revealed that the maximal amplitude intensity at the recording sites of the PU-PEDOT:PSS interface-modified electrode exceeded that of the PEDOT:PSS interface-modified electrode by 1.63 times, while the mean electrical signal amplitude increased from 55 μV to 78 μV [43]. Martinez-Cartagena et al. recorded neuronal cell electrical signals using EDOT–pyrrole-coated electrodes, demonstrating that the altered electrodes exhibited heightened sensitivity to bioelectrical signals with a superior SNR and nearly double the signal amplitude compared to conventional Ag/AgCl electrodes [44]. This highlights the efficacy of modifying the surface of implanted electrodes with enhancers in altering the compatibility of the electrode surface with biological tissues and enhancing the electrode’s capacity to capture physiological electrical signals. The incorporation of DMPC-SPIONs at electrode sites can substantially enhance the quality of the obtained electrophysiological signals, paving the way for a promising future as cost-effective and efficient bioelectrodes.

## 5. Conclusions

PEG and PEI were synthesized to modified iron oxide nanoparticles, which we have named PEG/PEI. These nanoparticles were then conjugated with DMPC to form DMPC-SPION nanoparticles. Subsequently, a revolutionary DMPC-SPION neuroelectrode was created by immobilizing these nanoparticles onto a nickel–chromium alloy electrode using an advanced electrochemical workstation. The results of this study revealed that the DMPC-SPION nanoparticles, with an average particle size of 12.75 ± 3.22 nm and Fe_3_O_4_ as the predominant crystalline phase, possess exceptional material properties and are biocompatible. The DMPC-SPION neuroelectrodes exhibited remarkable electrochemical characteristics, with a 91.4% increase in charge capacity and a significant 13% decrease in impedance. Furthermore, this cutting-edge neuroelectrode demonstrated outstanding capability in recording electrical signals, with peak potentials reaching as high as 171 μV and an impressive signal-to-noise ratio of 15.92. This groundbreaking DMPC-SPION neuroelectrode not only enhances biological and electrical functionalities significantly but also paves the way for advancements in neuroscience.

## Figures and Tables

**Table 1:** Intracellular Fe content (pg/cell) of PC-12 cells co-cultured.

Group	Iron Content (pg/Cell)
Control	0.58 ± 0.16
PEG/PEI-SPIONs	12.74 ± 3.14
DMPC-SPIONs	23.36 ± 7.9

## Data Availability

Data are contained within the article.
